# Developing mini-HTA to support evidence-based decision making in the Norwegian health services

**DOI:** 10.1017/S0266462326103894

**Published:** 2026-09-04

**Authors:** Helene Arentz-Hansen, Hanne Marie Rostad, Vidar Jusnes Vang, Anna Stoinska-Schneider, Maria Bjerk

**Affiliations:** 1 Norwegian Institute of Public Health, Norway; 2 The Norwegian University of Science and Technology, Norway; 3 Østfold Hospital, Norway; 4 Norwegian Medical Products Agency, Norway

**Keywords:** mini-HTA, hospital-based HTA, specialist health services, municipal healthcare services, Health Technology Assessment, Decision-making, Evidence-based healthcare

## Abstract

Health technology assessment (HTA) has become an essential tool for supporting evidence-based decision making in health care systems worldwide. Comprehensive HTA reports, however, are resource-intensive and time-consuming, making them impractical for many decisions that need to be made at the local or institutional level. This gap has led to the development of more pragmatic approaches that aim to balance methodological rigor with feasibility. In Norway, this challenge was addressed through the introduction of mini-HTA, which is a structured, simplified, and context-specific decision-support tool to inform local or institutional decisions about introducing new technologies, procedures, or organizational innovations. Mini-HTA provides a standardized framework for evaluating clinical effectiveness, safety, organizational impact, ethical considerations, and economic consequences while remaining feasible for clinicians and managers to apply in practice. Several key insights emerge from our experience with developing and implementing mini-HTA in Norway. First, adapting HTA approaches to local decision-making contexts increases feasibility and uptake, particularly where time and resources are limited. Second, governance structures, including clear allocation of responsibilities and leadership engagement, are crucial for the consistent use of mini-HTA in practice. Third, the Norwegian experience illustrates how a shared but flexible tool can be applied across different levels of care, including both hospitals and municipalities, while still allowing for contextual adaptation. Finally, the use of mini-HTA as a platform for collaboration across sectors highlights its potential not only as a decision-support tool, but also as a mechanism for coordinating priorities and responsibilities across organizational boundaries.

## Introduction

Norway has a universal health care system based on the principle of equal access regardless of socioeconomic status or geographic location. The system is semi-decentralized: 357 municipalities are responsible for primary health and long-term care services, including general practitioners, home care, rehabilitation and preventative services, and nursing homes, while four regional health authorities manage specialist care, including hospitals and advanced treatments. Specialist health services are financed by the state through block grants and activity-based funding and operate within fixed regional budgets that must cover the costs of implementing new interventions and running services. Municipal health and care services are financed mainly through local taxes and state transfers and are delivered within fixed municipal budgets (1). Yet both municipal and specialist healthcare services face the same fundamental challenges of limited resources and increasing demands. This creates a strong need for systematic, transparent, and evidence-informed decision-making processes to ensure fair and efficient allocation of resources. Across both municipal and specialist healthcare services, decision making is guided by three nationally endorsed priority-setting criteria: severity of the condition, expected health benefit, and resource use (2). These principles are anchored in national policy and regulations, including the Patients’ and Users’ Rights Act and the Regulation on Priority Setting (3). However, operationalization of these principles varies between health care sectors.

In 2008, the Norwegian Knowledge Centre for Health Services (NOKC), now the Norwegian Institute of Public Health (NIPH), was commissioned by the Western Norway Regional Health Authority to develop a system for mini-HTA in hospitals, comprising a structured assessment tool, defined roles and processes, and support functions. The basis for this commission was the follow-up of the National Health Plan (2007─2010) (4), which required the regional health authorities in Norway to review and improve decision-making processes related to local investments in high-cost medical equipment and pharmaceuticals, with the aim of establishing standardized procedures.

Then, in 2013, the Ministry of Health and Care Services established a national system for the introduction of new interventions in the specialist health service, called “New Methods.” *New Methods* is a national system for determining which interventions may be adopted in the specialist health service and functions as a prioritization tool under the responsibility of the four regional health authorities. The system operates along two tracks: **(1) National decisions**, which apply to all regional health authorities nationwide and typically concern whether an intervention should be introduced – or phased out – in Norwegian hospitals. These decisions primarily rely on national HTAs and cover all drugs for the public specialist health service, as well as selected medical devices and procedures. **(2) Local decisions**, which usually apply to individual hospitals. This track builds directly on the mini-HTA system developed from 2008 onwards, using a structured mini-HTA tool to support evidence-based decisions and clarify the implications of introducing – or discontinuing – interventions at the local level. Mini-HTAs typically evaluate new procedures, medical devices, and diagnostic methods, in addition to some organizational measures.

**Figure 1.**
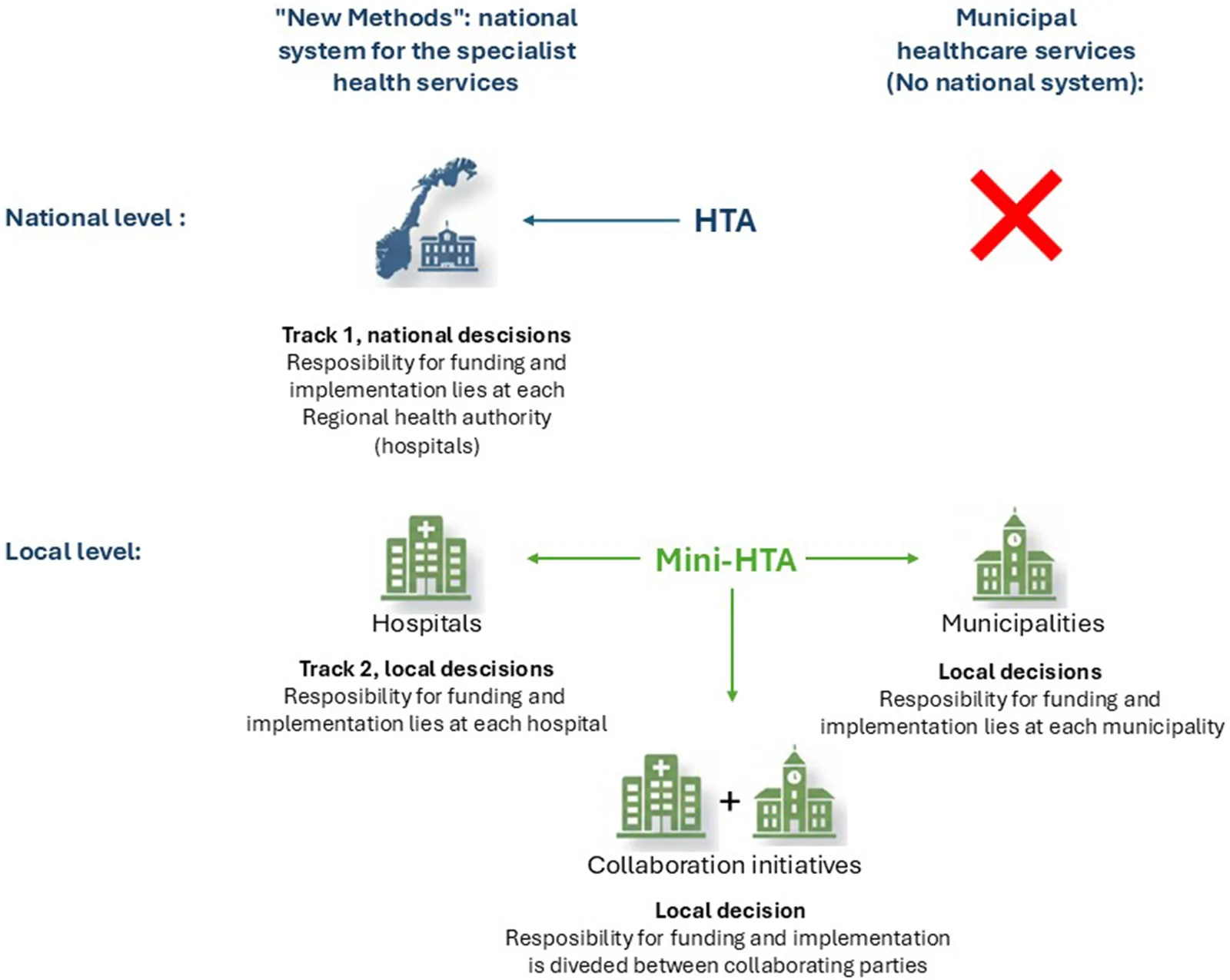
The use of HTA across levels and sectors in Norwegian healthcare.

A national track for decision making as established in the specialist health service (track 1) does not currently exist at the municipal level. However, a local track (track 2) has recently been developed for municipalities, inspired by hospital mini-HTAs, and work is ongoing to further develop this approach (Figure 1).

**Figure 2.**
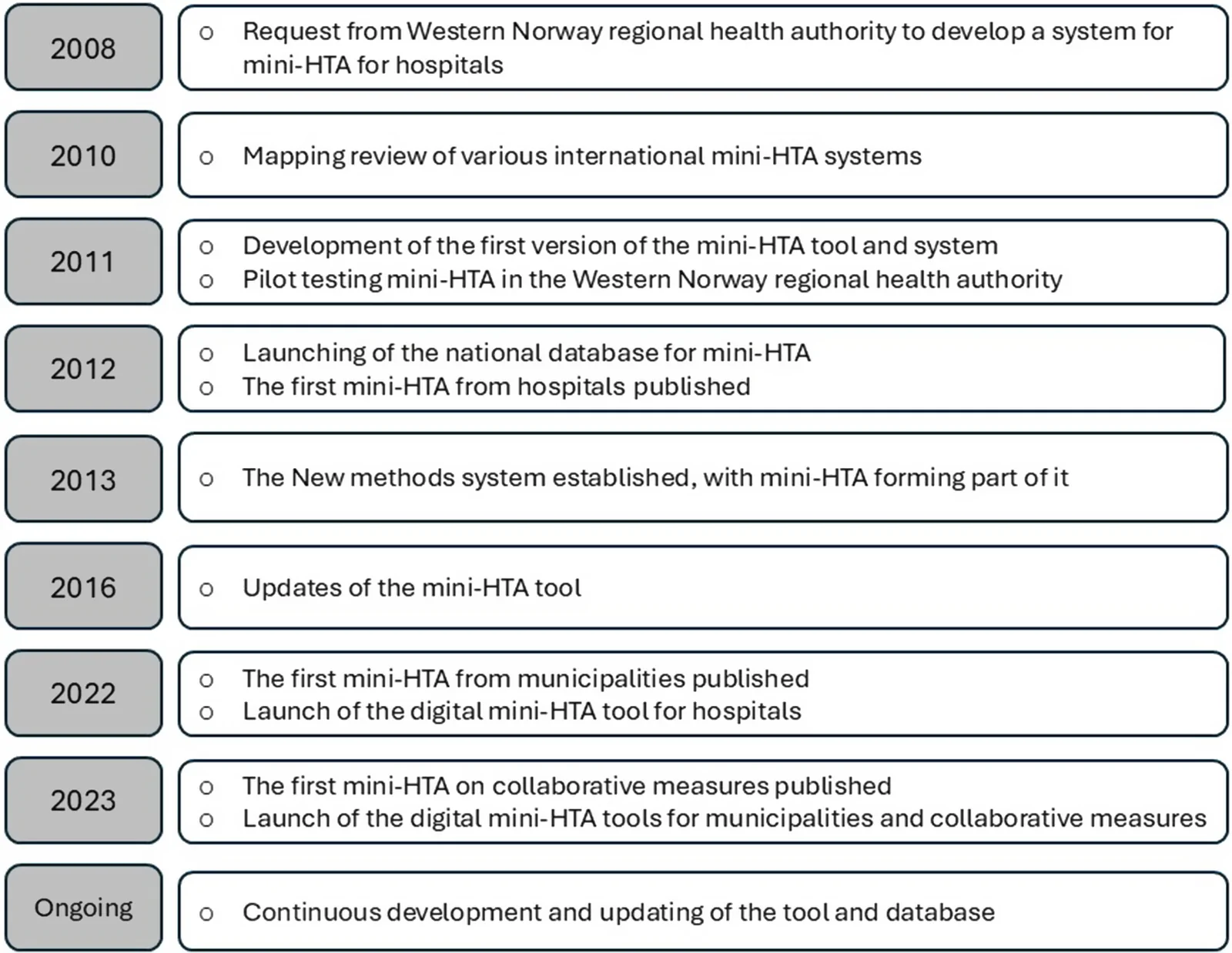
A timeline of the steps in the development of the mini-HTA tool and system.

Main milestones in the development of the mini-HTA system in Norway are indicated in Figure 2.

In this commentary, we describe and reflect on the development of mini-HTA in the specialist health service (track 2), and the opportunities and challenges in extending this approach to the municipal level. We aim to contribute practical, experience-based insights to the ongoing discussion on achieving decision making across all levels of care that is well-founded, systematic, coherent, and transparent, and ensuring equitable prioritization of health care services.

## Developing a mini-HTA system for local decision making in the specialist health services

**Figure 3.**
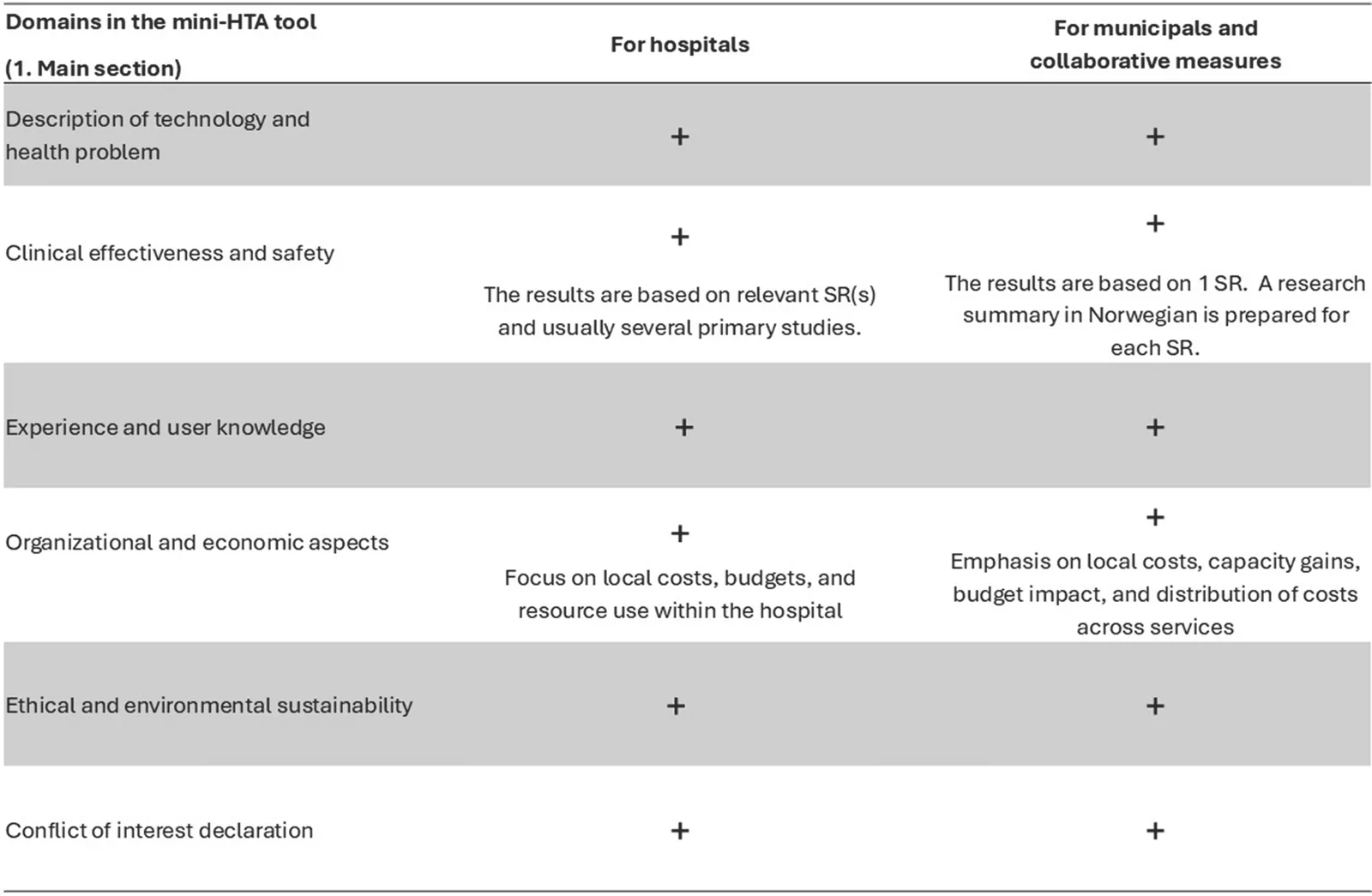
The main sections in the mini-HTA tools for hospitals, municipalities, and collaborative measures.

Following the 2008 commission, we mapped international mini-HTA systems and identified eight approaches across Europe, Australia, and North America (5). These highlighted the use of structured assessment tools and key elements for implementation, including management involvement, clear responsibilities, support functions, and transparency. The insights gained from the mapping review (5) were important in developing the mini-HTA system adapted to the Norwegian hospital context. A working group, including representatives from the NIPH and the Western Norway regional health authority, developed a proposal for both the assessment tool (mini-HTA tool) and the supporting organizational set-up for mini-HTA (2011) (6). The mini-HTA tool was developed as a structured three-part assessment form: (1) a main section presenting the knowledge base and assessment of consequences, (2) a peer review section, and (3) a final section providing recommendations for decision makers. The first and main section was developed based on the Danish, Canadian, and Australian mini-HTA tools (5). The content of this section includes the same core elements as a traditional HTA (7), but in a downscaled form and with a stronger emphasis on local context and feasibility. It includes a description of the assessed intervention and the relevant patient population, an evaluation of clinical effectiveness and safety based on evidence from research and systematic literature searches, ethical considerations, organizational and economic consequences, and implications for other stakeholders (Figure 3). Hence, it represents a structured, simplified, and context-specific basis relevant to decision making within hospitals. This section is completed by an internal working group at the hospital, including the proposer (often a clinician), a librarian, and an economist. Section 2, designated for the peer reviewer, consists of a checklist intended to verify whether the mini-HTA has been conducted satisfactorily. The mini-HTA is often carried out by clinicians who are advocating for the introduction of the intervention in question into the hospital, making independent peer review an important mechanism to ensure objectivity and balance. The only requirement for selecting peer reviewers is that they must be independent experts from another equivalent professional environment. The final and third section is intended to provide recommendations to the decision maker(s). This section is completed by the person responsible for preparing the case for decision making within the hospital – although the specific role and responsibilities may vary between hospitals – and provides an overall assessment of whether the available information is sufficient to support implementation of the intervention. Section 3 is optional and only for internal use at the relevant hospital. The mini-HTA tool also includes a declaration of interest to be filled in by both the proposer and the peer reviewer.

Since its introduction in 2011, more than 140 hospital mini-HTAs have been published. The mini-HTA tool has undergone informal evaluations and continuous refinements based on experience from these assessments. This iterative process has led to ongoing improvements in both content and format, while the overall structure has remained unchanged. These developments were further informed by participation in the European research project AdHopHTA (Adopting Hospital Based Health Technology Assessment in EU, 2012–2015; http://www.adhophta.eu/), which confirmed the relevance of the core domains in the Norwegian mini-HTA tool and highlighted the need to include political and strategic considerations. These aspects were subsequently incorporated into the final section of the tool (part 3) (8).

### Mini-HTA as an alternative to national HTA

In the *New methods* system, only a subset of interventions (other than pharmaceuticals) is intended to be assessed at the national level (track 1). For interventions that do not meet the criteria for a national assessment, but where uncertainties remain regarding effectiveness, safety, costs, or organizational consequences, the mini-HTA was included as a pivotal part of the system (track 2) (Figure 1). Whereas national HTAs are intended to inform overarching national decisions and adopt a more generalizable, system-level perspective, mini-HTAs are more context-specific, focusing on local organization, capacity, and implementation, and are therefore better suited to support hospital-level decision making. In addition, unlike the process for interventions proposed at the national level (track 1), there is no central coordination mechanism for “assessment proposals” at the hospital level (mini-HTA; track 2). Here, the individual hospitals themselves decide which interventions they want to conduct mini-HTA on, at what time, and how they plan the process. This lowers the barrier to assessing new interventions by reducing both the resource demands and the time required. However, the challenge is that the quality of mini-HTAs – and thus the decision-making basis – may vary, both because the methodological requirements for a mini-HTA are lower than for national HTAs, and because the individuals who carry out the assessments often have limited knowledge of HTA.

### The responsibilities for mini-HTA are shared among actors in the specialist health services

A white paper (9) specifies the allocation of tasks related to mini-HTA among relevant actors, that is, the NIPH, the hospitals, and regional health authorities. NIPH serves as the national resource group for mini-HTA with key responsibilities, including updating and refining the mini-HTA tool, managing the support function, and further development of the mini-HTA database. In addition, they provide training and methodological support, as well as advisory support to hospitals on their mini-HTA activities. Today, this group consists of a leader who is a researcher with HTA competence, a health economist, a librarian, and a web editor. An advantage of positioning the resource group outside the structure of the regional health authorities is that it enables it to support all hospitals equally and act as a neutral body. However, this organizational independence may also create a certain distance from the clinical settings where mini-HTAs are implemented. This distance can limit insight into local contexts, needs, and challenges, and may reduce the group’s ability to closely support implementation processes. To mitigate this, the resource group maintains regular dialogue with hospitals using mini-HTA. However, reaching hospitals with lower levels of engagement, that is, those that use the mini-HTA infrequently or have not fully integrated it into their decision-making processes, remains a challenge.

The hospitals are responsible for conducting mini-HTAs and applying them in decisions about the implementation or discontinuation of interventions. The regional health authorities are responsible for providing support services to ensure that hospitals have access to the necessary expertise to conduct mini-HTAs. Such support functions include, among others, methodological support, access to research librarians, assistance with health economic evaluations, and support for the analysis of budgetary implications. In the regions South-Eastern Norway Regional Health Authority and Western Norway Regional Health Authority, regional competence services for HTA have been established to support the mini-HTA work in their healthcare facilities. Such support has not been established in the other two regional health authorities in Norway. Our observations indicate that regional competence centers contribute positively to the production and publication of mini-HTAs. Regions with active centers show consistent output, while hospitals without such support tend to publish very few, if any, mini-HTAs.

### Applying the mini-HTA concept in municipal healthcare services and across sectors

From 2020 to 2023, the NIPH conducted a pilot project, A Knowledge System for Municipalities, in collaboration with the Western Norway University of Applied Sciences. We developed and tested a scalable model for knowledge support in the municipal cluster Knowledge Municipality Health Care West (10). The model was designed to help municipalities systematically identify and use research evidence in service planning and decision making. An important outcome in this project was that we found that municipalities required a broader decision-making basis than research evidence alone could provide. This prompted us to also test the mini-HTA concept in the municipal sector, with the aim of translating the mini-HTA tool for hospitals into municipal settings characterized by different responsibilities, resources, and decision-making structures.

While the specialist health service has had more than a decade to build routines, competence, and culture around structured evidence use, municipalities are only now beginning this journey. The transfer of the mini-HTA concept from hospitals to municipal care is therefore not merely an administrative extension – it represents an important shift in how evidence and prioritization principles may be operationalized across levels of care. The development of a system for knowledge support for the municipal health and care services has been highlighted as an important governmental initiative, included in white papers such as “Benefit, Resource, and Severity – Prioritization in the Health and Care Services” (11). Although there are numerous existing regional and national competence centers, there is often a lack of local collaboration structures in the municipal sector that promote the actual use of research as a basis for decision making.

### Adapting the mini-HTA tool and process to municipal health care

**Figure 4.**
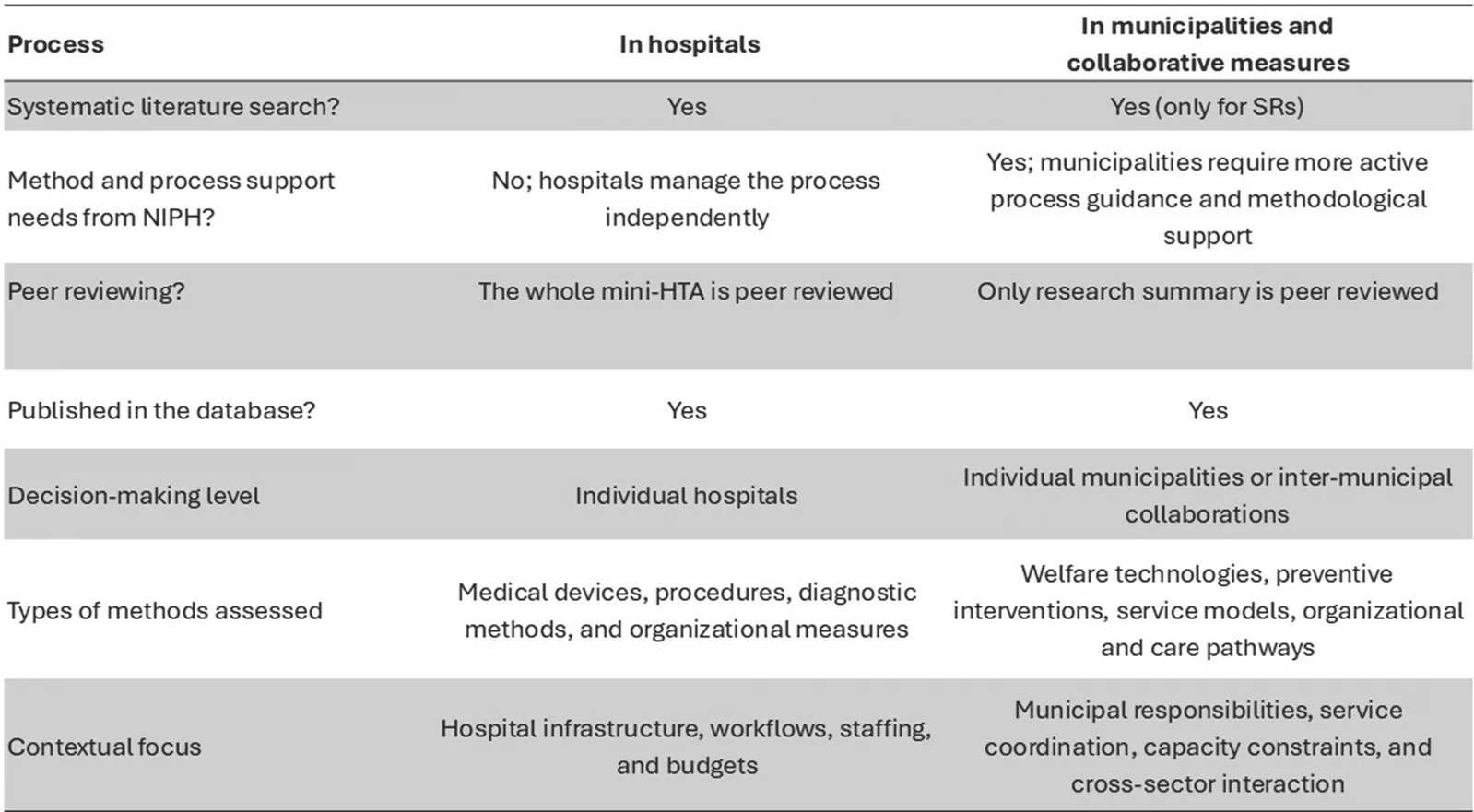
Comparison of mini-HTA processes in hospitals, municipalities, and collaborative settings.

The adaptation of the mini-HTA concept to the municipal context began in 2022, and since then 23 mini-HTAs have been conducted in collaboration with municipalities, including six joint assessments involving both hospitals and one or more municipalities. The wide range of topics addressed, from welfare technologies, school health services to integrated care, illustrates the breadth and diversity of decision-making needs at the municipal level (Figure 4). Experience from these assessments highlighted both similarities and important differences compared with the specialist health service. While the core principles of the mini-HTA were retained, feedback from municipalities prompted targeted adaptations of the tool. These included the introduction of a section on environmental impact, revisions to the economic section to better capture capacity gains, and a simplified evidence component based on a single systematic review. Some of these elements, such as environmental considerations, are also relevant for hospitals and were therefore incorporated into the hospital mini-HTA tool. This illustrates how experiences from the municipal context can feed back into and strengthen the development of the mini-HTA tool more broadly, enhancing its applicability and relevance across levels of care (Figure 3).

The process of conducting a mini-HTA differs compared to the hospitals (Figure 4). In the municipalities, access to librarians and scientific resources is scarce, and employees have limited time and resources to perform development work. In addition, decision-making needs tend to be more heterogeneous, often spanning multiple services, both within a single sector and across sectors, such as health, education, and social care. This requires consideration of a broader range of stakeholders, outcomes, and contextual factors than is typically the case in hospital-based assessments.

A municipal mini-HTA starts with an identified knowledge-need from the municipality. For each project, an interdisciplinary team from the NIPH, usually comprising one or two researchers, an economist and a librarian, works together with municipal representatives, often including managers or advisors. The work needs to be anchored with municipal leadership before starting up, and is carried out over ~3 months, including five structured digital workshops. In these processes, the NIPH provides process guidance and is responsible for the structure and content of the workshops, in addition to conducting the sections on research and economy. The municipalities are responsible for completing the remaining parts of the mini-HTA form. The outcome is a complete mini-HTA published in the mini-HTA database.

### Extending mini-HTA on collaborative measures

Many interventions span multiple levels of the health care system, involving both specialist and municipal services. Decisions on such interventions raise additional questions compared to single-sector initiatives, particularly related to shared responsibility, coordination of implementation, budgetary implications, and the distribution of costs and benefits across levels of care.

To address these, the NIPH saw a need to develop a dedicated mini-HTA tool specifically designed for collaborations between municipalities and hospitals, where they work together within the same mini-HTA tool. When working together across municipal and hospital care, it is essential to have a shared process and tool for optimal implementation and scaling, budget implications, and cost distribution between the hospital and municipality upon implementing the assessed initiative. Like the mini-HTAs conducted in municipalities, the NIPH provides process support for the team of employees from both the hospital and participating municipality(-ies). Our experience is that the mini-HTA can function not only as a local decision tool, but also as a governance mechanism for managing shared responsibilities and priorities across organizational boundaries.

## The national mini-HTA database as an enabling infrastructure

The establishment of a national database for mini-HTA, to consolidate all completed assessments, was one of the recommendations from the Danish initiative (6). However, in contrast to their approach, we decided that this database should be open access to promote transparency and facilitate the reuse of mini-HTAs, thereby avoiding duplication of work. The website www.minimetodevurdering.no was launched in 2012 and named “The National Database for Mini-HTA.” The database is a central component of the mini-HTA system. Since its inception in 2012, it has undergone continuous development and now houses all mini-HTAs from hospitals, municipalities, and collaborative initiatives. The latest major upgrade (2022/23) involved the digitization of the three mini-HTA tools for hospitals, municipalities, and collaborative efforts, so that they became an integrated part of the database. An important goal of digitizing the mini-HTA tools was to facilitate cooperation among participants, which is particularly crucial when hospitals and municipalities collaborate on a joint mini-HTA. We have addressed this by, for example, allowing multiple users to simultaneously work within the tool, even if they are in different locations, and providing fields for exchanging comments during the process for those working on the tool.

## Key lessons learned and further development

- Ensuring relevance: development, collaboration, and future adaptation of mini-HTA

An important experience from our work with developing the mini-HTA concept so far has been the importance of involvement and close collaboration with stakeholders in the hospitals and municipalities. For mini-HTA to serve as an effective tool, it is crucial to obtain information regarding hospitals and municipalities’ specific needs, processes, and contextual factors regarding decision making and prioritization. Combining their user input with our expertise in HTA provides a solid basis for continuous development of a useful mini-HTA tool.

We had to take several considerations into account when we developed the mini-HTA tools – for hospitals, municipalities, and collaborative measures. First, it was important to determine the appropriate level of comprehensiveness, that is, the tools must provide a sufficient basis for decision making in hospitals and municipalities, while still being feasible for busy clinicians and staff to complete. Second, the tools had to be applicable for the assessment of a wide variety of interventions, spanning from technologies to service models or care processes. Third, the tools had to be adaptable for use in various types of hospitals, including both university and local hospitals, and municipalities of varying sizes and capacities. And fourth, the tools had to be manageable and user-friendly for people lacking HTA expertise. For mini-HTA in hospitals, it even had to be manageable without guidance from the resource group at NIPH.

The mini-HTA tools will remain under continuous refinement. The aim is not to complete a “final” version of the tool, but rather to maintain it as a flexible tool that evolves in line with the emerging needs of the health care system. As the volume and diversity of new interventions available to the health services increase, including digital interventions, personalized medicine, and technologies involving artificial intelligence (AI), the tool must adapt to these changes to remain relevant, useful, and feasible for both hospitals and municipalities. Moreover, the use of AI to streamline the mini-HTA process will be important. The most time-consuming aspect for clinicians in hospitals in conducting mini-HTAs is extracting data from research papers into the main section of the mini-HTA, and AI integration here could be a natural starting point.

1. Building bridges between health services

As stated in the Government’s White Paper (Meld. St. 9 (2023–2024)) (12), it is an explicit goal of the Norwegian government to view the healthcare system as an integrated system rather than as separated into specialist and municipal health services. We therefore consider it important that mini-HTAs from both hospitals and municipalities are published in a shared database. The same White paper emphasizes the importance of collaboration between the levels in the health services. In the mini-HTA processes including both hospitals and municipalities, we have observed that the mini-HTA meetings function as a collaborative platform for equitable engagement, with NIPH acting as a “neutral” facilitator. Our vision is therefore that these mini-HTA processes will facilitate mutual understanding and foster improved collaboration across health care levels.

1. Importance of commitment and structural differences in health services

Commitment from management and leadership is essential to the governance and effective use of mini-HTA in hospitals and municipalities. Governance concerns how decision-making authority and responsibilities are organized and how leadership engagement embeds mini-HTA in formal decisions. As hospitals and municipalities differ in their organizational structures, implementation and governance of mini-HTA must be adapted accordingly. In the specialist health service, mini-HTA work can be facilitated and coordinated from the regional level. In the municipalities, such clear structures do not exist, leaving each municipality to decide whether to adopt mini-HTA as a decision-support tool. To address this challenge, we collaborate with other overarching organizations and structures. For our mini-HTA work with municipalities, we liaise with the Municipal Collaboration Arena for Research (KSF), a national platform that fosters cooperation between municipalities, research institutions, and other stakeholders to strengthen knowledge development in local services. For mini-HTA initiatives with hospital–municipal collaboration, we engage with the Health Collaboratives, formal partnerships between hospitals and municipalities designed to improve coordination and integrated care across service levels.

1. Cultural differences

Hospitals have traditionally had a longer-standing culture of research and evidence-informed decision making than municipalities. While municipal employees increasingly seek research to inform key decisions, both access to relevant evidence and the competence, routines, and resources needed to apply this evidence remain more limited, particularly in smaller municipalities. This constrains the development of an evidence-seeking culture, and the capacity to critically appraise research literature at the level required for mini-HTA. Different types of competencies among employees in the municipalities and in hospitals, as well as differences in resources and personnel allocated to research-related tasks, mean that mini-HTAs can be carried out independently in hospitals, whereas municipalities have so far relied on guidance from the NIPH. Yet, a commonality of all mini-HTAs is that ownership of the content and results rests with those conducting the assessments, that is, the municipalities and hospitals.

## Moving forward: understanding the role of mini-HTA across levels of care

In this commentary, we have described and reflected on the development of the mini-HTA tool and system in hospitals and its subsequent adaptation in municipalities, as well as on collaborative measures. Building on the substantial experience gained with the implementation of mini-HTA, we have identified a need to examine how mini-HTA functions in practice. This raises questions about how mini-HTA is currently used in hospitals and municipalities, including its level of implementation and the factors that may influence its uptake. In settings where mini-HTAs are produced regularly, it is of particular interest to explore how the tool is integrated into decision-making processes, for example, in relation to procurement, prioritization, and the management of opportunity costs within constrained local budgets. Furthermore, it remains unclear how the mini-HTA framework is followed up and supported within national governance structures, including its role within the New Methods system. Gaining further insight into these aspects may help clarify how mini-HTA can contribute to more informed and consistent decision making across the health system.

## Conclusion

The Norwegian experience highlights several key contributions to the HTA field. Mini-HTA demonstrates how HTA methodologies can be adapted to support decision making beyond national-level assessments, particularly in decentralized health systems. It also illustrates the importance of governance, leadership, and organizational embedding for successful implementation. Furthermore, extending mini-HTA across levels of care shows its potential to support more coordinated and consistent decision making in integrated health systems.
